# Supplementary material for: Antidementia Effects of Alternanthera philoxeroides in Ovariectomized Mice Supported by NMR-Based Metabolomic Analysis
Source: Molecules. 2021 May 9;26(9):2789. doi: 10.3390/molecules26092789 (PMC8125874; doi:10.3390/molecules26092789)
Supplement: Supplementary file 1 [file molecules-26-02789-s001.zip › molecules-1199224-supplementary.pdf]

Supplementary Materials

Antidementia Effect of *Althernanthera philoxeroides* in Ovariectomized Mice Supported by NMR-Based Metabolomic Analysis.

Charinya Khamphukdee [1*], Orawan Monthakantirat [2], Yaowared Chulikhit [2], Chantana Boonyarat [2], Supawadee Daodee [2], Possatorn Aon-im [1], Juthamart Maneenet [2], Yutthana Chotritthirong [3], Prathan Luecha [1], Nazim Sekeroglu [4] Anake Kijjoa[5*]

1 Division of Pharmacognosy and Toxicology, Faculty of Pharmaceutical Sciences, Khon Kaen University, Khon Kaen, 40002, Thailand; charkh@kku.ac.th (C.K.); possa.torn@kkumail.com (P.A.); prathanl@kku.ac.th (P.L.)

2 Division of Pharmaceutical Chemistry, Faculty of Pharmaceutical Sciences, Khon Kaen University, Khon Kaen, 40002, Thailand; yaosum@kku.ac.th (Y.C.); chaboo@kku.ac.th (C.B.); csupawad@kku.ac.th (S.D.); juthamart_pp@hotmail.com (J.M.)

3 Graduate School of Pharmaceutical Sciences, Khon Kaen University, Khon kaen 40002, Thailand; yutthana_ck@kkumail.com (Yu.C.)

4 Department of Horticulture, Faculty of Agriculture, Kilis 7 Aralik University, 79000 Kilis, Turkey; Nsekeroglu@gmail.com (N.S.).

5 ICBAS-Instituto de Ciências Biomédicas Abel Salazar and CIIMAR, Universidade do Porto, Rua de Jorge Viterbo Ferreira, 228, 4050-313, Porto, Portugal (A.K).

*Correspondence: ankijjoa@icbas.up.pt. Tel.: +351-220428331 (A.K.), charkh@kku.ac.th (C.K.)

1.. Statistical analysis of the Ethanol Crude Extract of A. philoxeriodes on OVX-induced cognitive deficits-like Behavioral

Table S1. One-way analysis of variance (ANOVA) test of the morris water maze task

Group comparison	ANOVA followed by Tukey's post hoc test	
	P	F ($DF_{between\ group}$, $DF_{residual}$)
Sham VS. OVX Vehicle-treated	< 0.05	F(4,44)=12.639
OVX VS. OVX+E_2 (1 µg/kg/day)	< 0.001	
OVX VS. OVX+AP250 (250 mg/kg/day)	0.05	
OVX VS. OVX+AP500 (500 mg/kg/day)	< 0.001	
OVX+AP250 VS. OVX+AP500	<0.001	

Table S2. One-way analysis of variance (ANOVA) test of novel objective recognition test (NORT)

Group comparison	ANOVA followed by Tukey's post hoc test	
	P	F ($DF_{between\ group}$, $DF_{residual}$)
Sham VS. OVX Vehicle-treated	< 0.001	F(4,41)=11.570
OVX VS. OVX+E_2 (1 µg/kg/day)	< 0.001	
OVX VS. OVX+AP250 (250 mg/kg/day)	< 0.001	
OVX VS. OVX+AP500 (500 mg/kg/day)	< 0.001	

Table S3. One-way analysis of variance (ANOVA) test of Y-maze test

Group comparison	ANOVA followed by Tukey's post hoc test	
	P	*F* ($DF_{between\ group}$, $DF_{residual}$)
Sham VS. OVX Vehicle-treated	< 0.001	*F*(4,45)=11.844
OVX VS. OVX+E_2 (1 µg/kg/day)	< 0.001	
OVX VS. OVX+AP250 (250 mg/kg/day)	< 0.05	
OVX VS. OVX+AP500 (500 mg/kg/day)	< 0.001	

2. Statistical analysis of the Ethanol Crude Extract of A. philoxeriodes on OVX-induced lipid peroxidation in the brain

Table S4. One-way analysis of variance (ANOVA) test of lipid peroxidation in mice whole brain

Group comparison	ANOVA followed by Tukey's post hoc test	
	P	*F* ($DF_{between\ group}$, $DF_{residual}$)
Sham VS. OVX Vehicle-treated	< 0.001	*F*(4,18)=10.024
OVX VS. OVX+E_2 (1 µg/kg/day)	< 0.001	
OVX VS. OVX+AP250 (250 mg/kg/day)	< 0.001	
OVX VS. OVX+AP500 (500 mg/kg/day)	< 0.001	

3. Statistical analysis of of the Ethanol Crude Extract of A. philoxeriodes Ameliorates on OVX-induced Changes in neuroinflammatory cytokines and PI3K/AKT pathway -related gene in mRNA Expressions of Mice Hippocampus and Frontal Cortex

Table S5. One-way analysis of variance (ANOVA) test of IL-1β mRNA in hippocampus

Group comparison	ANOVA followed by Tukey's post hoc test	
	P	*F* ($DF_{between\ group}$, $DF_{residual}$)
Sham VS. OVX Vehicle-treated	< 0.05	*F*(3,16))=13.344
OVX VS. OVX+E_2 (1 µg/kg/day)	< 0.001	
OVX VS. OVX+AP250 (250 mg/kg/day)	< 0.05	
OVX VS. OVX+AP500 (500 mg/kg/day)	< 0.05	

Table S6. One-way analysis of variance (ANOVA) test of IL-1β mRNA in frontal cortex

Group comparison	ANOVA followed by Tukey's post hoc test	
	P	*F* ($DF_{between\ group}$, $DF_{residual}$)
Sham VS. OVX Vehicle-treated	< 0.05	*F*(3,22)=7.46.
OVX VS. OVX+E_2 (1 µg/kg/day)	< 0.05	
OVX VS. OVX+AP250 (250 mg/kg/day)	< 0.05	
OVX VS. OVX+AP500 (500 mg/kg/day)	< 0.05	

Table S7. One-way analysis of variance (ANOVA) test of IL-6 mRNA in hippocampus

Group comparison	ANOVA followed by Tukey's post hoc test	
	P	F ($DF_{between\ group}$, $DF_{residual}$)
Sham VS. OVX Vehicle-treated	< 0.05	F(3,17)=7.079
OVX VS. OVX+E_2 (1 µg/kg/day)	< 0.05	
OVX VS. OVX+AP250 (250 mg/kg/day)	< 0.05	
OVX VS. OVX+AP500 (500 mg/kg/day)	< 0.05	

Table S8. One-way analysis of variance (ANOVA) test of IL-6 mRNA in frontal cortex

Group comparison	ANOVA followed by Tukey's post hoc test	
	P	F ($DF_{between\ group}$, $DF_{residual}$)
Sham VS. OVX Vehicle-treated	< 0.05	F(3,22)=7.046
OVX VS. OVX+E_2 (1 µg/kg/day)	< 0.05	
OVX VS. OVX+AP250 (250 mg/kg/day)	< 0.05	
OVX VS. OVX+AP500 (500 mg/kg/day)	< 0.05	

Table S9. One-way analysis of variance (ANOVA) test of TNF-α mRNA in hippocampus

Group comparison	ANOVA followed by Tukey's post hoc test	
	P	F ($DF_{between\ group}$, $DF_{residual}$)
Sham VS. OVX Vehicle-treated	< 0.05	F(3,18)=20.534
OVX VS. OVX+E_2 (1 µg/kg/day)	< 0.001	
OVX VS. OVX+AP250 (250 mg/kg/day)	< 0.001	
OVX VS. OVX+AP500 (500 mg/kg/day)	< 0.001	

Table S10. One-way analysis of variance (ANOVA) test of TNF-α mRNA in frontal cortex

Group comparison	ANOVA followed by Tukey's post hoc test	
	P	F ($DF_{between\ group}$, $DF_{residual}$)
Sham VS. OVX Vehicle-treated	< 0.05	F(3,17)=27.219
OVX VS. OVX+E_2 (1 µg/kg/day)	< 0.001	
OVX VS. OVX+AP250 (250 mg/kg/day)	< 0.001	
OVX VS. OVX+AP500 (500 mg/kg/day)	< 0.001	

Table S11. One-way analysis of variance (ANOVA) test of PI3K mRNA in hipoocampus

Group comparison	ANOVA followed by Tukey's post hoc test	
	P	F ($DF_{between\ group}$, $DF_{residual}$)
Sham VS. OVX Vehicle-treated	< 0.001	F(3,16)=20.32
OVX VS. OVX+E_2 (1 µg/kg/day)	< 0.001	
OVX VS. OVX+AP250 (250 mg/kg/day)	Not significant	
OVX VS. OVX+AP500 (500 mg/kg/day)	< 0.05	

Table S12. One-way analysis of variance (ANOVA) test of PI3K mRNA in frontal cortex

Group comparison	ANOVA followed by Tukey's post hoc test	
	P	*F* ($DF_{between\ group}$, $DF_{residual}$)
Sham VS. OVX Vehicle-treated	< 0.05	*F*(3,16)=11.304
OVX VS. OVX+E_2 (1 µg/kg/day)	< 0.001	
OVX VS. OVX+AP250 (250 mg/kg/day)	Not significant	
OVX VS. OVX+AP500 (500 mg/kg/day)	< 0.001	
OVX+AP250 VS. OVX+AP500	< 0.05	

Table S13. One-way analysis of variance (ANOVA) test of AKT mRNA in hipoocampus

Group comparison	ANOVA followed by Tukey's post hoc test	
	P	*F* ($DF_{between\ group}$, $DF_{residual}$)
Sham VS. OVX Vehicle-treated	< 0.05	*F*(3,16)=10.451
OVX VS. OVX+E_2 (1 µg/kg/day)	< 0.001	
OVX VS. OVX+AP250 (250 mg/kg/day)	Not significant	
OVX VS. OVX+AP500 (500 mg/kg/day)	< 0.05	

Table S14. One-way analysis of variance (ANOVA) test of AKT mRNA in frontal cortex

Group comparison	ANOVA followed by Tukey's post hoc test	
	P	*F* ($DF_{between\ group}$, $DF_{residual}$)
Sham VS. OVX Vehicle-treated	< 0.05	*F*(3,16)=5.513
OVX VS. OVX+E_2 (1 µg/kg/day)	< 0.05	
OVX VS. OVX+AP250 (250 mg/kg/day)	Not significant	
OVX VS. OVX+AP500 (500 mg/kg/day)	< 0.05	

4. Serum metabolic profile of the OVX mice-treated with the crude ethanol extract of A. philoxeroides.

Table S15. List of all metabolites that found in ^{1}HNMR spectra of serum samples.

No.	Chemical shift (ppm)	multiplicity	STOCSY	p-value	Metabolite
1.	0.96174	t	0.96174 (t), 1.71 (m), 3.76 (t)	$1x10^{-14}$	Leucine
2.	0.9950	d	0.99505 (d), 1.047 (d), 2.277 (dh), 3.621 (d)	$1x10^{-15}$	Valine
3	1.0152	d	0.9372 (t), 1.0152 (d), 1.259 (m), 1.997 (m), 3.682 (d)	$1x10^{-13}$	Isoleucine
4.	3.4439	dd	1.149 (d), 3.4439 (dd), 3.549 (dd), 3.872 (m)	$1x10^{-15}$	Propylene glycol
5.	1.3352	d	1.3352 (d), 4.11 (q)	$1x10^{-15}$	Lactate
6.	1.4852	d	1.4852 (d), 3.781 (q)	$1x10^{-12}$	Alanine
7.	1.931	s	1.931 (s)	$1x10^{-12}$	Unknow1
8.	2.1493	m	2.1493 (m), 2.643 (t), 3.842 (dd)	$1x10^{-13}$	Methionine
9.	2.3822	s	2.3822 (s)	$1x10^{-16}$	Pyruvate

10.	2.4165	d	2.4165 (s)	$1x10^{-16}$	Succinate
11.	2.5352	s	2.5352 (d)	$1x10^{-16}$	Unknow2
12.	2.7643	t	2.7643 (s)	$1x10^{-16}$	Butanedinitrile
13.	3.2438	s	3.2438 (t), 6.887 (d), 7.20 (d)	$1x10^{-14}$	Tyramine
14.	3.2724	dd	3.2724 (s)	$1x10^{-14}$	Betaine
15.	3.0490	s	3.049 (s), 4.046 (s), 3.244 (dd), 3.402 (s), 3.463 (m), 3.352 (dd), 3.713 (m)	$1x10^{-10}$	Creatinine
16.	3.489	m	3.244 (dd), 3.402 (m), 3.463 (m), 3.52 (dd), 3.713 (m), 3.829 (m), 3.901 (dd), 4.654 (d)	$1x10^{-12}$	Alpha-D-glucose
17.	3.6552	s	3.538 (m), 3.655 (m), 3.781 (m)	$1x10^{-11}$	Glycerol
18.	3.9425	dd	3.9425 (s)	$1x10^{-10}$	Glycorate
19.	3.9651	m	3.769 (dd), 3.9651 (m)	$1x10^{-15}$	Serine
20.	4.0663	dqt	3.212 (s), 3.528 (m), 4.0663 (m)	$1x10^{-9}$	Choline
21.	4.2588	s	1.335 (d), 3.621 (d), 4.2588 (dqt)	$1x10^{-15}$	Threonine
22.	6.5324	d	6.5324 (s)	$1x10^{-15}$	Fumarate
23.	6.9031	s	3.026 (dd), 3.945 (dd), 6.9031 (d), 7.198 (d)	$1x10^{-15}$	Tyrosine
24.	7.0872	dd	7.837 (s), 7.0872 (s), 3.99 (dd), 3.259 (dd)	$1x10^{-15}$	Histidine
25.	7.3344	s	7.427 (d), 7.3344 (dd), 6.903 (d)	$1x10^{-17}$	Protocatechuic acid
26.	8.4672	s	8.4672 (s)	$1x10^{-15}$	Formate
27.	3.3733	s	3.3733 (s)	$1x10^{-15}$	Unknow3

Table S16. Summary of OPLS-DA metabolic changes in control (DI water) and AP500 group ^{1}HNMR spectra.

"+" indicate higher correlation in AP500 group, whereas "-" indicates higher correlation in Control group (DI water). "*" indicate p-value less than 0.05, "**" indicates p-value less than 0.01 and "***" indicates p-value less than 0.001.

Metabolite	Chemical shift	OPLS-DA model (Control Vs. AP500)
Isoleucine	0.9372 (t), 1.0152 (d), 1.259 (m), 1.997 (m), 3.682 (d),	-0.6469*
Lactate	1.3352 (d), 4.11 (q)	-0.5542*
Butanedinitrile	2.7643 (s)	-0.6896**
Tyramine	3.2438 (t), 6.887 (d), 7.20 (d)	0.3439*
Betaine	3.2724 (s)	0.7486**
Propylene glycol	1.149 (d), 3.4439 (dd), 3.549 (dd), 3.872 (m)	0.7618***
Alpha-D-glucose	3.244 (dd), 3.402 (m), 3.463 (m), 3.52 (dd), 3.713 (m), 3.829 (m), 3.901 (dd), 4.654 (d)	0.3439**
Glycerol	3.538 (m), 3.655 (m), 3.781 (m)	0.7711***
Threonine	1.335 (d), 3.621 (d), 4.2588 (dqt)	-0.5379*

Table S17. Summary of the pathway analysis related with metabolic of AP500 group obtained from the MetPA.

Pathway name	Math status	p	-log(p)	Holm p	Impact
Galactose metabolism	2/27	0.002995	2.5235	0.25162	0.02924
Glycerolipid metabolism	1/16	0.052072	1.2834	1.0	0.23676
Fructose and mannose metabolism	1/18	0.058425	1.2334	1.0	0.0
Glycolysis/ Gluconeogenesis	1/26	0.083499	1.0783	1.0	0.000084
Glycine, serine and threonine metabolism	1/34	0.10804	0.96643	1.0	0.04655
Amino sugar and nucleotide sugar metabolism	1/37	0.1171	0.93144	1.0	0.0
Tyrosine metabolism	1/42	0.13204	0.87928	1.0	0.02463

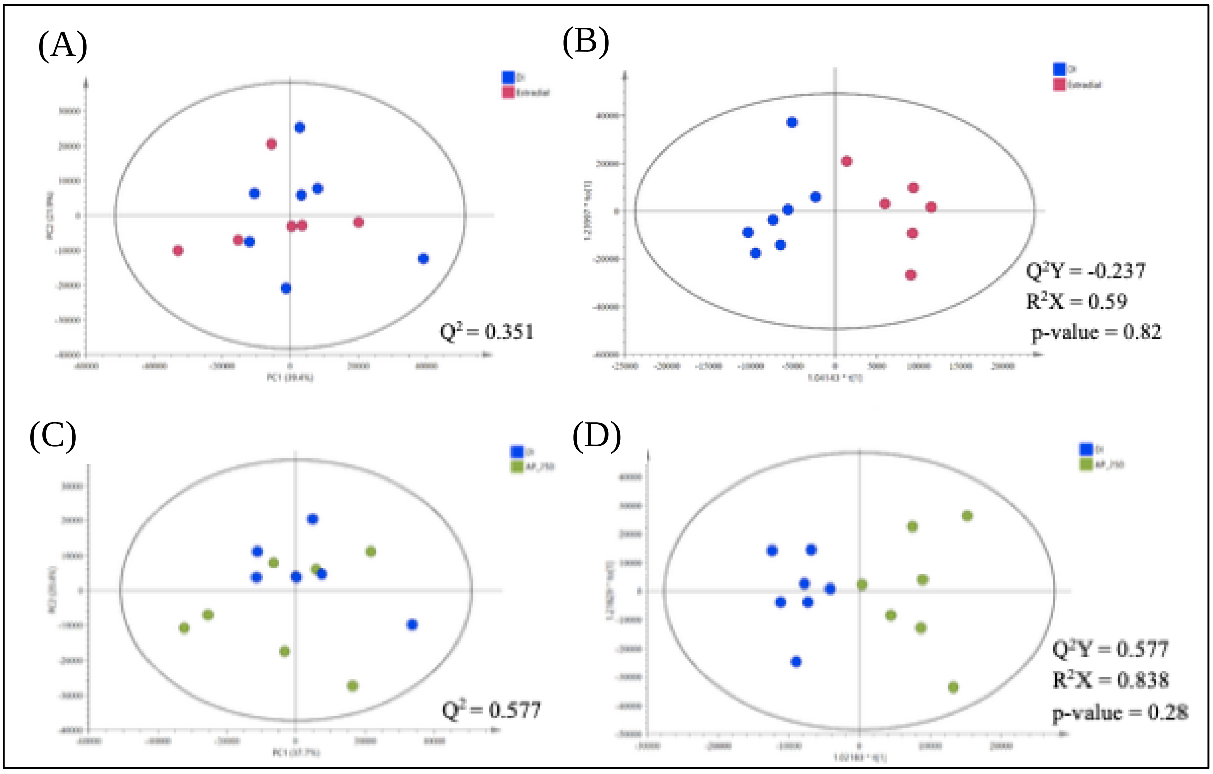

Figure S1. The PCA and OPLS-DA score plot. PCA score plot between control (DI water) and 17β-estradiol group (A). OPLS-DA score plot between control (DI water) and 17β-estradiol group (B). PCA score plot between control (DI water) and AP 250 (C). OPLS-DA score plot between control (DI water) and AP 250 (D)
